# Field Emission Properties of Cu-Filled Vertically Aligned Carbon Nanotubes Grown Directly on Thin Cu Foils

**DOI:** 10.3390/nano14110988

**Published:** 2024-06-06

**Authors:** Chinaza E. Nwanno, Arun Thapa, John Watt, Daniel Simkins Bendayan, Wenzhi Li

**Affiliations:** 1 Department of Physics, Florida International University, Miami, FL 33199, USA; cnwan007@fiu.edu (C.E.N.); dsimk005@fiu.edu (D.S.B.); 2 Center for Integrated Nanotechnologies, Los Alamos National Laboratory, Los Alamos, NM 87545, USA

**Keywords:** VACNTs, copper, PECVD, field emission

## Abstract

Copper-filled vertically aligned carbon nanotubes (Cu@VACNTs) were grown directly on Cu foil substrates of 0.1 mm thicknesses at different temperatures via plasma-enhanced chemical vapor deposition (PECVD). By circumventing the need for additional catalyst layers or intensive substrate treatments, our in-situ technique offers a simplified and potentially scalable route for fabricating Cu@VACNTs with enhanced electrical and thermal properties on thin Cu foils. Comprehensive analysis using field emission scanning microscopy (FESEM), transmission electron microscopy (TEM), energy-dispersive X-ray spectroscopy (EDS) mappings, and X-ray diffraction (XRD) revealed uniform Cu filling within the VACNTs across a range of synthesis temperatures (650 °C, 700 °C, and 760 °C). Field emission (FE) measurements of the sample synthesized at 700 °C (S700) showed low turn-on and threshold fields of 2.33 V/μm and 3.29 V/μm, respectively. The findings demonstrate the viability of thin Cu substrates in creating dense and highly conductive Cu-filled VACNT arrays for advanced electronic and nanoelectronics applications.

## 1. Introduction

Since their discovery in 1991 by Ijima [1], CNTs have been extensively researched for possible applications in myriads of fields. They exhibit exceptional chemical stability, high aspect ratio, low work function, large field enhancement factor, superior mechanical strength, and excellent electrical and thermal conductivities, making them promising materials for field emission applications [2,3,4,5,6,7]. VACNTs, a special kind of CNT, consist of individual CNTs aligned perpendicular to the substrate [8,9,10]. Although they share similar alignment with the three-dimensional (3D) vertically aligned graphene nanosheets (VAGNAs) comprising interconnected graphene sheets arranged vertically relative to the substrate and forming a porous, 3D network [11,12,13], both materials have marked differences. For instance, the individual CNTs forming the VACNTs possess hollow cylindrical (tube-like) structures characterized by a high aspect ratio, whereas the VAGNAs retain the planar shape (sheet-like) structure of the component graphene sheets while providing a large surface area. The hollow nature of the CNTs provides a unique advantage over the 3D VAGNAs for applications requiring material encapsulation, allowing the CNTs to be filled with other foreign materials. Studies have suggested that due to the hollow cavities of the CNTs, filling them with foreign materials will drastically improve their intrinsic properties and enhance their performances in field emission devices and several other nanoscale applications, including nanosensors [14], nanomagnets [15], nanoswitches [16], nanothermometers [17], batteries [18], supercapacitors [19], etc.

The resulting filled CNTs (X@CNTs) display the characteristic features of both the host CNTs and the foreign filler materials, together with elongated cavities acting as templates to generate 1D nanostructures such as nanowires [20]. For instance, studies have shown that CNTs filled with ferromagnetic materials (Fe, Co, and Ni) display excellent magneto-resistance and, thus, can be used as nanomagnets in magnetic data storage devices [21,22,23,24]. The CNTs ensure durability and stability by protecting the ferromagnetic filler from possible oxidation. In field emission devices, the electrical and thermal conductivities of CNTs are greatly improved by encapsulating highly conductive nanowires inside the cores of the CNT arrays. This greatly increases the field enhancement factor and reduces the turn-on and threshold fields. In 2021, our group reported very low turn-on and threshold fields of 1.57 V/µm and 2.43 V/µm, respectively, and a high field enhancement factor (ß = 3061) for Cu-filled VACNTs (Cu@VACNTs) grown on bulk Cu disks [25]. These excellent field emission properties resulted from synergetic contributions from the VACNTs and the highly conductive encapsulated Cu nanowires.

Depending on whether filling occurs during or after CNT growth, the techniques are classified into two broad categories: ex-situ and in-situ. The ex-situ techniques, which include the sonication-assisted wet chemical method [26], supercritical fluid chemical deposition (SFCD) [27], the solution infusion method [28], the chemical fluid deposition method (CFD) [29], and the electrochemical deposition method [30], involve encapsulating foreign materials into the hollow cavities of CNTs post CNT synthesis. On the other hand, in-situ filling occurs simultaneously with CNT growth. The arc-discharge [31] and chemical vapor deposition (CVD) [32] methods are the most used in-situ methods. Meanwhile, unlike the ex-situ techniques, which involve multiple steps that require opening the closed ends of CNTs, the in-situ methods present a single-step synthesis route for filled CNTs (X@CNTs) with well-preserved capsules and closed ends [33].

Several foreign materials, including metals [34,35,36] and metal oxides [37,38,39], organic molecules [40,41,42], carotenes [43,44,45], fullerenes [46,47,48], fluorescent NPs [49], etc., have been used to fill CNTs for several applications. Among these materials, Cu-filled CNTs (Cu@CNTs) have gained huge recognition owing to their excellent electrical and thermal conductivities, the low cost of Cu, and weak interaction with carbon. Furthermore, studies have demonstrated that the inherent high conductivity of CNTs can be increased by encapsulating Cu inside the core of the CNTs [50,51,52]. Despite these impressive results, the filling methods employed are fraught with several issues, such as poor yield of filled CNTs, multiple intricate steps, improper alignment of the resulting Cu@CNTs, and the need for Cu salts.

Furthermore, to achieve Cu@CNTs via the in-situ technique, the CNTs need to be grown directly on the Cu substrates without any extra catalyst layers. However, reports have shown that it is challenging to synthesize CNTs on Cu because of its poor catalytic activity and extremely low carbon solubility [53]. Cu has occupied 3d orbitals, which prohibit the formation of covalent bonds with hydrocarbon molecules. The small binding energy of Cu with carbon also suppresses CNT graphitization during growth [54]. Atthipalli et al. [55] grew multi-walled CNTs (MWCNTs) on bulk Cu wafers but by first depositing Ni film as a catalyst layer. Similarly, Yin et al. [56] reported bamboo-like CNTs on an oxygen-free Cu substrate by first sputtering Ni particles as catalysts on the Cu substrate. Rao et al. [57] also reported CNT growth on TEM Cu grids coated with Ni. Sepahvand et al. [58] obtained dense VACNT arrays by depositing Ni and chromium (Cr) as both catalyst and barrier layers, respectively. Meanwhile, it is important to point out that prior to the catalyst deposition, an intermediate buffer layer is first deposited onto the substrate material to prevent the diffusion of the catalyst particles into the Cu substrates. Aside from this being a tortuous procedure, the presence of the intermediate layer increases the contact resistance between the CNTs and the Cu substrates. In addition, it is impossible to achieve in-situ filling of the CNTs with Cu following the procedure described above because the CNTs grow on the deposited catalyst particles and are more likely to be filled with them. There are a few successful reports on the direct synthesis of MWCNTs on Cu substrates by first activating the catalytic properties of the Cu substrates by either acid or sulfur treatment for many hours [53,59,60]. However, this process is long and expensive. Our group has tried to address these intricacies by developing a facile in-situ method for filling VACNTs with Cu [25]. Although the technique obviates the deposition of extra layers and acid treatment, the Cu@VACNTs were grown directly on thick Cu disks (thickness = 0.6 mm), which are not good for practical micro- and nanoelectronics applications.

Herein, we report the direct synthesis of Cu@VACNTs on thin Cu foils (thickness = 0.1 mm) at different temperatures. Generally, compared to thick substrates, it is more difficult to grow CNTs on their thinner counterparts because they lack the structural integrity to withstand the high mechanical and thermal stress during the CNT growth process. They crack, melt, or undergo other structural deformations, which could in turn impact the uniformity and quality of the resulting CNTs.

## 2. Experimental Section

### 2.1. Cu@VACNTs Synthesis

Arrays of copper-filled VACNTs (Cu@VACNTs) were synthesized on 0.1 mm thick Cu foil substrates using the direct current (DC) PECVD method described in our previous report [61]. The Cu foil substrates were ultrasonicated with acetone and isopropyl alcohol for 10 min each to eliminate organic and inorganic contaminants. After cleaning, the substrates were allowed to dry in the open air before being transferred to the PECVD chamber for CNT growth.

The growth chamber was pumped down to a base pressure of 0.01 Torr. Subsequently, ammonia (NH_3_) gas was introduced at a flow rate of 110 sccm, maintaining a constant chamber pressure of 7 Torr to create the reduced environment necessary for CNT growth. Under these conditions, the Cu substrates were heated to growth temperatures of 650 °C, 700 °C, and 760 °C at a rate of 50 °C/min. Uniform catalytic active sites were formed on the substrate surface due to the etching effect of NH_3_ on the substrate at elevated temperatures. These catalytic sites were necessary for the nucleation of CNTs. Upon reaching the desired growth temperature, the DC plasma was turned on, and the power was maintained at 70 W. This was immediately followed by introducing acetylene (C_2_H_2_) as the carbon precursor gas at a constant flow rate of 30 sccm. The system was turned off after 30 min of growth and was allowed to cool down at the base pressure. The samples of Cu@VACNTs synthesized at 650 °C, 700 °C, and 760 °C were named as S650, S700, and S760, respectively.

### 2.2. Material Characterization

A field emission scanning electron microscope (FESEM) was used to characterize the surface structure of the as-synthesized Cu@VACNTs at an accelerating voltage of 15 kV. The XRD patterns of the different samples were obtained using the Siemens Diffractometer D5000 (Munich, Germany) with Cu Kα radiation (λ=1.54 Å). The nanostructures of the as-synthesized Cu@VACNTs were examined using an image-aberration-corrected ThermoFisher Titan 80–300 (Waltham, MA, USA) fitted with an EDAX Octane Elite T solid-state X-ray spectrometer operated at 300 kV. The Cu@VACNT samples were prepared for TEM by gently scrapping them off the Cu foil substrate with a surgical blade and flushed off onto the TEM grid by dropping alcohol on the blade. Low-magnification images were taken to reveal the continuity of the Cu filling inside the CNTs. High-magnification images of the CNT shell and the filled Cu core were taken to show their structures and interfaces. TEM-EDS mapping was also carried out to confirm the chemical constituent of the guest material inside the cores of the CNTs.

### 2.3. FE Measurement

As shown in the Electronic Supplementary Information (ESI), Figure S1, a diode configuration was used to measure the field emission properties of the sample S700 Cu@VACNTs in a vacuum chamber with a base pressure of approximately 1 × 10^−6^ Torr. Using a silver paste, the cathode was prepared by gluing the as-synthesized Cu@VACNTs onto the stainless-steel plate. The anode was a solid cylindrical stainless-steel rod with a 0.803 cm^2^ diameter. The separation distance between both electrodes was maintained at 615 μm with the aid of ceramic and plastic spacers. Fifty-volt increments of voltage bias were applied using a DC power source (Matsusada AU-15P20, Otsu, Japan), and the emission current was measured with a Keithley 236 unit. At least five cycles of current density vs. electric field (J vs. E) characteristics were recorded with multiple samples to ensure the reproducibility of data.

## 3. Results and Discussion

### 3.1. VACNT Structure

**Figure 1:**
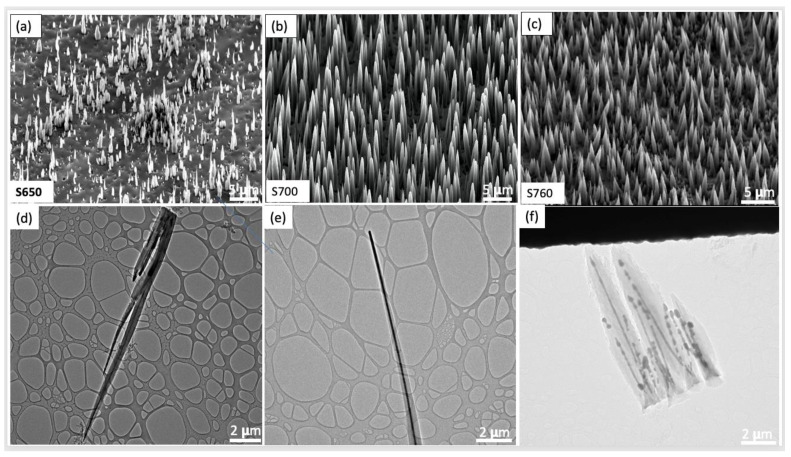
(**a**–**c**) SEM images of VACNTs synthesized at 650 °C, 700 °C, and 760 °C at a constant pressure of 7 Torr, plasma power of 70 W, and NH_3_ and C_2_H_2_ flow rates of 110 sccm and 30 sccm, respectively, for 30 min. (**d**–**f**) Corresponding TEM images of (**a**–**c**).

The FESEM images in Figure 1a–c reveal the effect of different growth temperatures on the morphology and density of the VACNT arrays. In Figure 1a**,** sparsely grown VACNTs of non-uniform height (between 0.4 and 10 μm), diameter (between 300 and 670 nm), and inter-tube spacings can be observed, some of which appear in bundles. The poor growth, non-uniformity, and bundled nature of these VACNTs can be attributed to the fact that the growth temperature (650 °C) was insufficient to break the surface of the Cu substrate into uniform and distinct nanosized islands for CNT growth. In addition, 650 °C may not be high enough to sufficiently dissociate the C_2_H_2_ into carbon atoms, leading to insufficient carbon species for further dissolution into the available nano islands. At a much higher synthesis temperature of 700 °C, the VACNTs appear as freestanding uniform VACNTs with average diameter and height of 940 nm and 14 μm, respectively (Figure 1b). At 760 °C (Figure 1c), the average diameter of the VACNTs was measured as 1.2 μm. Interestingly, the CNTs were slightly shorter than the S700 samples, with an average height of 8.5 μm. This might be attributed to the excessive carbon deposition at a higher temperature, which caused early growth termination. Also, most CNTs appear as bundles due to Ostwald ripening, which causes individual nanoparticles to coalesce and form bigger ones. As a result, numerous individual VACNTs grow from these bigger but single catalyst sites to form bundles [62].

Figure 1d–f show the corresponding TEM images of the VACNTs filled with foreign materials. Herein, it is evident that all the samples were filled with the guest material from their roots to the tips, creating core–shell structures. It can be seen that all the Cu@VACNT samples possess tapered structures with the number of graphitic layers decreasing from the roots to the tips. As a result, it can be said that the VACNT walls close to the tip of the Cu core were formed at the later stages of the synthesis process [63]. This implies that the growth procedure follows the tip growth mechanism described in our previous report [25], where the Cu tips serve as the catalytically active sites for the decomposition and precipitation of carbon atoms to form VACNT shells. Figure 1f shows that, although the individual VACNTs in the bundle are filled, the Cu nanowires are discontinuous and characterized by segments and dots. This can be attributed to the high internal energy from the very high growth temperature (760 °C). Consequently, the atomic activity of the Cu atoms in the nanowires increases. As a result, the Cu nanowires stretch due to pre-melting [64], decreasing the bonding strength of the Cu atoms and causing atomic gliding dislocations and grain boundary movement to occur [65].

**Figure 2:**
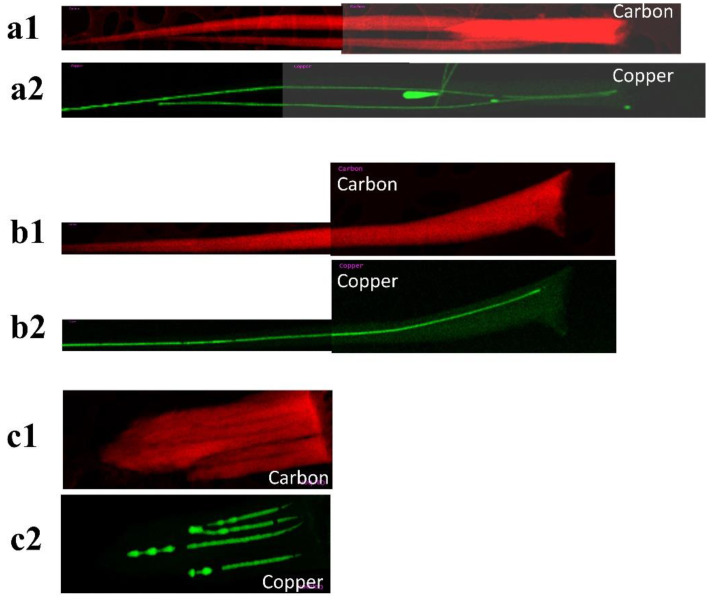
TEM-EDS mapping of (**a1**,**a2**) S650, (**b1**,**b2**) S700, and (**c1**,**c2**) S760 Cu@VACNTs.

The EDS mappings in Figure 2(a1–c2) reveal that the interior regions of darker contrast identified in the TEM images in Figure 1d–f are Cu nanowires. Figure 2(a1,a2) are the EDS mappings of two carbon nanotubes synthesized at a temperature of 650 °C; the two CNTs are in contact at their roots. Figure 2(a2) shows that the two carbon nanotubes are filled with continuous Cu nanowires. Figure 2(b1,b2) are the EDS mappings of a nanotube synthesized at a temperature of 700 °C; the nanotube is also filled with a long continuous Cu nanowire. From the mapping, we can also deduce that each of the individual VACNTs has Cu nanowires completely encapsulated in them. Figure 2(c1,c2) show the EDS mappings of a bundle of nanotubes synthesized at a temperature of 760 °C. Figure 2(c2) shows Cu nanowires’ discontinuity and Cu dots inside the carbon nanotubes.

**Figure 3:**
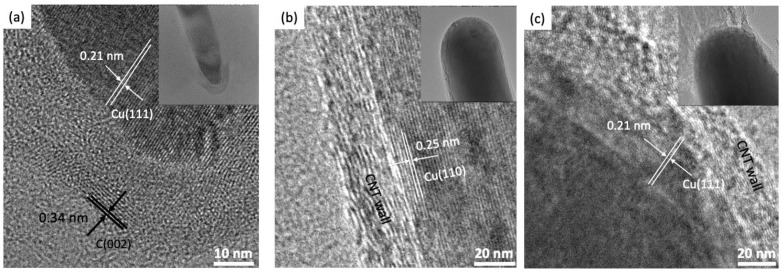
HRTEM images of (**a**) S650, (**b**) S700, and (**c**) S760 Cu@VACNTs. The insets provide zoomed-out views of the corresponding Cu@VACNTs.

Figure 3a–c are the HRTEM images of the various Cu@VACNT samples (the insets provide zoomed-out views of the corresponding Cu@VACNTs). In Figure 3a, two sets of regular crystal lattice spacings, 0.21 and 0.34 nm [66,67], related to the separation between two (111) planes of face-centered cubic (fcc) Cu crystal and (002) graphitic carbon planes are observed. Figure 3b,c indicate the presence of the (110) and (111) planes of the fcc Cu crystal with lattice spacings of 0.25 and 0.21 nm, respectively [66]. These observations demonstrate that the Cu nanowires encapsulated inside the S650, S700, and S760 Cu@VACNTs are single crystals with good crystallization.

**Figure 4:**
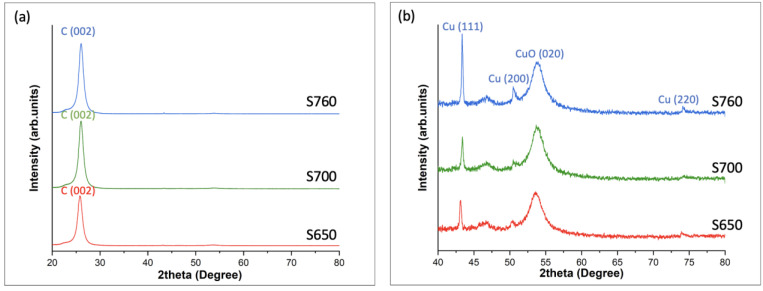
XRD patterns of the S650, S700, and S760 samples. (**a**) shows the peak of the graphitic carbon layers, and (**b**), zoom-in of (**a**), shows the peaks of different Cu crystals encapsulated inside the VACNTs.

Figure 4 shows the XRD pattern of the as-synthesized Cu@VACNTs at 600 °C, 700 °C, and 760 °C. Figure 4a shows diffraction peaks (2θ) for all samples at 26° related to the (002) graphitic planes. Figure 4b is the close-up view of Figure 4a in the 2θ range of 40–80°, it shows the diffraction peaks of pure Cu crystals at 43.18°, 50.34°, and 74.06°, which can be indexed as (111), (200), and (220) planes of the fcc Cu phase. However, we observed a broad diffraction peak at 52.30 related to the (020) crystalline phase of copper oxide (CuO). The observed CuO impurity could be from residual Cu nanoparticles attached to the surface of the VACNTs which are susceptible to oxidation upon exposure to atmospheric conditions. During the VACNT growth and Cu filling processes, not all Cu particles are fully encapsulated within the VACNTs. Some residual nanoparticles adhere to the external surfaces of the VACNTs due to surface energy interactions [68]. These surface-bound Cu nanoparticles, when exposed to air, readily oxidize to form CuO [69].

From these findings, it can be clearly seen that all the growth temperatures resulted in Cu-filled VACNTs, although their densities and structures are different. These results are slightly different from previous findings where we reported discrete Cu nanorod-filled CNTs at 650 °C [25]. Here, due to the thinness of the Cu foil, heating under the reduction environment was able to induce surface breakup of the Cu substrates even at a lower temperature of 650 °C. As a result, the disintegrated solid Cu nanoparticles were transformed into their quasi-liquid state, facilitating the filling of the VACNTs via quasi-liquid capillary adsorption [70,71].

### 3.2. Field Emission (FE) Measurements

#### 3.2.1. Field Emission Theory

Electrons from the surfaces of metals or semiconductors can only be knocked off when an external force supplies additional energy [72]. This extra energy can be produced using a variety of methods, including thermal processes, energy storage in an electric field, using the kinetic energy of charges, or light energy. Depending on the type of source, there are four primary ways to eject electrons from the solid surfaces: (1) cold emission, (2) heated emission, (3) field emission (FE), and (4) secondary emission. The FE method, which involves electron emission from a conductive metal surface by applying a strong electric field, takes advantage of sharp electrodes to enhance the local electric field [73]. In contrast to the other three techniques, the FE process transfers energy to trapped electrons in the material by deforming the potential barrier on its surface.

**Figure 5:**
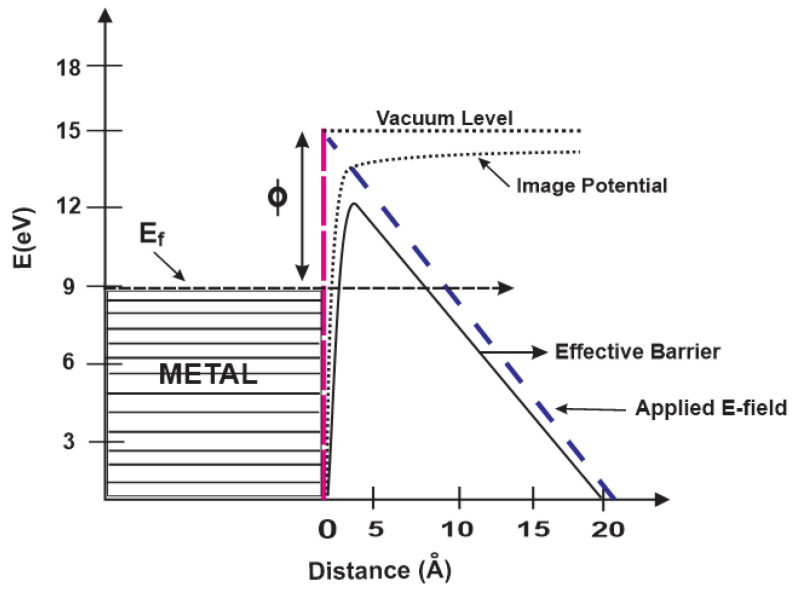
Potential energy diagram showing how an exterior electric field affects the energy barrier for electrons at the surface of a metal.

When an external electric field is applied, the potential barrier changes depending on the strength of the field. Figure 5 illustrates the potential as a simple plane with the electron needing enough energy to exit the material. This energy is known as the work function of the metal and is typically expressed in electron volts (eV). The red dashed line depicts the potential barrier before applying an external electric field. When an electric field is applied (blue dashed line), this potential barrier is distorted, which is shown as a curved dashed line (image potential) in the image. The stronger the applied electric field, the more the barrier is lowered and narrowed (effective barrier) and as a result, cold-trapped electrons near the Fermi energy (E_F_) level can escape into the vacuum by the ‘quantum tunneling’ effect, leading to field emission [74].

With the advancement of micro and nanofabrication technology in recent decades, generating high electric fields has been made possible by using sharp cathodes with few-hundred-nanometer tip diameters. However, compared to conventional metal emitters, CNTs possess a smaller tip curvature from which electrons are extracted. The reduced tip diameters in CNTs translate to a larger field enhancement factor and a greatly reduced turn-on field. In addition to the nanosized tips of the CNTs, their other properties, such as high mechanical strength, high electronic and heat conductivities, chemical inertness, and high aspect ratio, contribute to the excellent FE properties exhibited by CNTs. The FE behavior strongly depends on the CNTs’ morphology, spatial distribution, diameter, degree of alignment, the contact resistance between the CNTs and the substrate, and the nature of the CNTs’ tips [75].

#### 3.2.2. Field Emission Results

**Figure 6:**
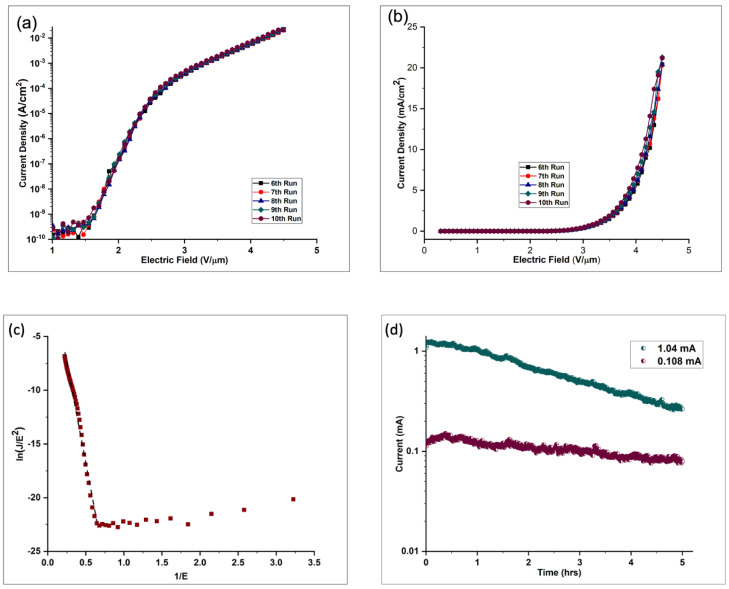
Graph of emission current density vs. applied electric field (J vs. E plot) of the S700 sample, (**a**) semi-log and (**b**) linear scale. (**c**) Fowler–Nordheim (F-N) plot of the S700 sample. (**d**) Stability test of the S700 sample at initial currents of 0.108 mA and 1.04 mA.

The emission currents from the Cu@VACNTs were measured at different electric fields to understand the performance of the Cu@VACNT emitters. Figure 6 presents the FE results from this study. To ensure reproducible emission characteristics, a ‘cleaning conditioning’ procedure was conducted during the first five current density vs. applied electric field (J vs. E) cycles [76]. The conditioning process evaporates surface absorbates from the VACNTs, which could otherwise trigger the FE quicker than their absorbate-free counterparts, resulting in instabilities and significant fluctuations in the measured emission current densities. Thus, the FE results presented in Figure 6a,b were from the subsequent five cycles after the initial cleaning process. Figure 6a,b are the semi-log and linear scale representations of the J vs. E properties of the Cu@VACNTs, respectively. From the curves of the J vs. E plots in Figure 6a, we estimated the turn-on (E_turn-on_) and threshold (E_th_) electric fields. E_turn-on_ was defined as the applied electric field required to obtain an emission current density of 10 μA/cm^2^ and E_th_ as the applied electric field required to produce an emission current of 1 mA/cm^2^. The Cu@VACNTs showed low E_turn-on_ and E_th_ values of 2.33 V/μm and 3.29 V/μm, respectively. Compared to the E_turn-on_ and E_th_ (1.77 V/μm and 2.43 V/μm) values previously reported by our group, the slightly larger E_turn-on_ and E_th_ reported in this work could be a result of increased screening effect, stemming from reduced inter-tube spacing between individual VACNT emitters and lower aspect ratio caused by the larger diameter of the VACNTs. From Figure 6b, the maximum emission current density was found to be in the range of 20.5 to 21.0 mA/cm^2^ at an applied electric field of 4.5 V/μm.

The emission current density of CNT emitters can be expressed by the following Fowler–Nordheim (F-N) equation [77]:

(1) $$J=\left(\frac{{A\beta }^{2}E^{2}}{\Phi }\right)\exp\left(\frac{-{B\Phi }^{\frac{3}{2}}}{\beta E}\right)$$

where J is the emission current density, A $(1.56\times 10^{-6}$ ${A\;V}^{-2}\;\mathrm{eV}$) and B (6.83 × 10^9^$\;{\mathrm{eV}}^{-\frac{3}{2}}$ ${\mathrm{Vm}}^{-1}$) are constants dependent on the work function of the material and the local electric field at the emission tip, Φ (5 eV) is the work function of the VACNT field emitters, E is the applied macroscopic electric field between the VACNT emitters and the anode, and β is the field enhancement factor. Equation (1) can further be transformed as follows:

(2) $$\mathrm{In}\left(\frac{J}{E^{2}}\right)=\mathrm{In}\left(\frac{{A\beta }^{2}}{\Phi }\right)-\frac{{B\Phi }^{\frac{3}{2}}}{\beta E}$$

From Equation (2), it can be seen that $\mathrm{In}\left(\frac{J}{E^{2}}\right)$ is linearly proportional to $\frac{1}{E}$ with a slope of $-\frac{{B\Phi }^{\frac{3}{2}}}{\beta }$ Hence, the field enhancement factor, β, can be calculated from the slope of the F-N plot shown in Figure 6c. The straight line in the F-N plot indicates the quantum mechanical tunneling process [78]. From the calculation, we obtained a high field enhancement factor of β =2037.

It is important to note that Equation (1) used in our analysis is a simple approximation of the F-N equation employed by Edgcombe et al. [79] in their study. While the F-N equation is theoretically derived for electron emission from flat surfaces at 0 K [74], it has been found to be experimentally valid at much higher temperatures. This is because field emission is primarily driven by the electric field, and the tunneling mechanism described by the F-N equation continues to be valid and dominant even when thermal effects are present. The simplified version used in our study omits the correction factor v(y), which accounts for exchange and correlation effects for very small tip diameters. Given the relatively large average diameter of our Cu@VACNTs compared to their length, we estimate that the impact of curvature corrections is minimal.

Additionally, the work function of 5 eV used for our analysis is a common value employed in field emission studies involving CNT emitters. This value is well established in the literature for pure CNTs and provides a standardized basis for comparison [80]. Although our CNTs are filled with Cu, which typically has a work function of 4.6 to 4.9 eV [81], the interaction between the Cu and the carbon matrix could alter the Fermi level and affect the overall work function [82]. Thus, choosing 5 eV provides a reasonable approximation, allowing us to maintain consistency with previous studies and ensure ease of comparison.

Stability is another essential factor to be considered in determining the applicability of CNTs as field emission devices. To assess this, we tested the stability of two Cu@VACNTs as shown in Figure 6d. At a low initial test current, the Cu@VACNTs showed good stability, decreasing from 0.108 mA to only about 0.079 mA, which represents about 26% current degradation from the initial test current after the 5 h test. At a higher initial test current of 1.04 mA, the Cu@VACNTs showed good stability in the first 1.5 h with only 19% degradation from the initial test current. Thereafter, the current degraded rapidly from 0.89 mA to a low of 0.264 mA, representing a total degradation of 74% from the initial test current after the 5 h test. The significant current degradation at elevated test current is likely due to joule heating effects exacerbated by the low inter-tube spacing within the arrays. In a closely packed VACNT array, individual VACNTs shield each other from the applied electric field due to electrostatic screening [83]. This leads to a reduction in the effective emission area and the field enhancement factor (β), as only a fraction of the VACNTs actively participate in the emission process [84]. When this happens, the active VACNT emitters experience increased localized heating (joule heating) impeding effective thermal dissipation. This effect becomes more pronounced at high emission currents as the resulting overheating weakens the bond between the VACNTs and the substrate, leading to the peeling of the VACNTs from the substrate surface [85].

**Figure 7:**
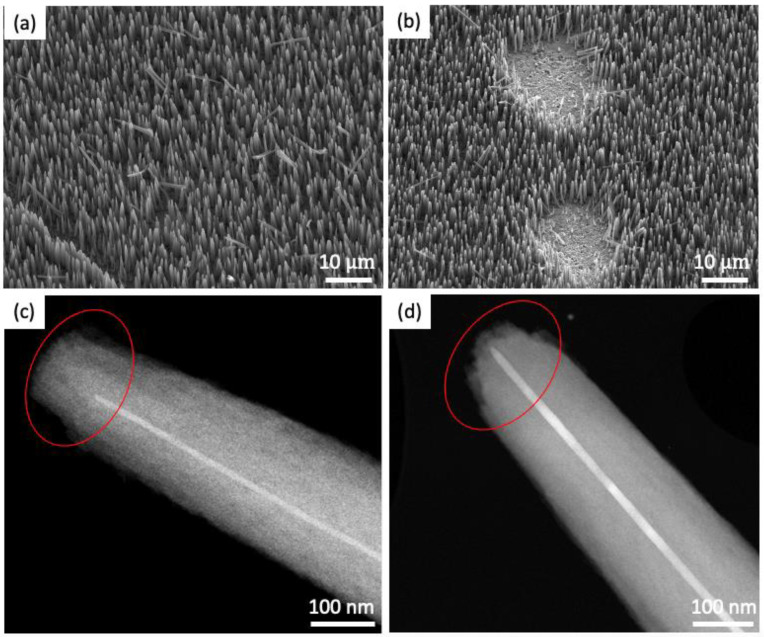
(**a**,**b**) SEM images of the VACNTs after emission stability tests at 0.108 mA and 1.04 mA, respectively. (**c**,**d**) TEM images showing the tips of VACNTs in Figures (**a**,**b**).

Figure 7a,b show the SEM images of the VACNTs after the stability tests at initial test currents of 0.108 mA and 1.04 mA, respectively. Figure 7b shows that more VACNTs were peeled off from the Cu substrate during the test at 1.04 mA compared to the VACNTs tested at the low current of 0.108 mA (Figure 7a). As discussed earlier, the observed degradation at the higher test current is most likely a result of the increased joule heating at 1.04 mA, exacerbating thermal stress within the VACNT array, and weakening the adhesion between the VACNTs and the substrate, hence the peeling. Figure 7c,d are the TEM images of samples presented in Figure 7a,b, respectively, showing the tip geometry change after the stability test. At the tip of the VACNTs, where the electric field is concentrated, the field emission can lead to the evaporation of carbon atoms [86]. This field-induced evaporation occurs because the strong local electric fields at the tips can overcome the cohesive energy of carbon atoms, causing them to be ejected from the tips. This process gradually reshapes the tip, leading to blunting [87]. Figure 7d reveals that the tip of the VACNT sample, subjected to a high test current of 1.04 mA, not only exhibited blunting but also suffered significant damage to its structural integrity. This deterioration is attributed to the stronger local electric fields and increased joule heating associated with higher test currents, accelerating the degradation of the tips, which serve as the primary emission sites.

## 4. Conclusions

In this study, we have successfully synthesized Cu-filled VACNT (Cu@VACNT) arrays on thin Cu foils of 0.1 mm thickness at different temperatures, via the PECVD technique. Our findings highlight the significant role of substrate thickness in promoting the Cu-filling process at lower temperatures. This was evident from the complete filling of the as-synthesized VACNTs with Cu at a temperature as low as 650 °C, which contrasts with our previous studies on thicker Cu substrates where filling occurred only at temperatures ranging from 700 to 750 °C. The detailed SEM and TEM analysis revealed a consistent core structure with the encapsulated Cu nanowires showing good crystallinity, despite the occurrence of some discontinuities and dislocations at a higher growth temperature of 760 °C. The field emission measurements of the S700 Cu@VACNTs indicated favorable field emission properties, with low turn-on and threshold fields. This underscores the huge potential of Cu@VACNT emitters in field emission applications due to the synergetic effect of the sharp tips of the VACNTs and the highly conductive Cu nanowire fillers. The stability test revealed satisfactory performance at a lower emission current, though challenges remain at a much higher emission current likely due to the increased joule heating effect caused by the low inter-tube spacing within the VACNT arrays. To fully utilize the potential of these Cu@VACNTs, further work is required to control the density of the VACNTs and optimize the inter-tube spacing for reduced screening effects.

## Data Availability

The data presented in this study are not available due to privacy.

## Supplementary Materials

The following supporting information can be downloaded at https://www.mdpi.com/article/10.3390/nano14110988/s1, Figure S1: Schematic illustration of the setup for field emission measurements.
