# Supplementary material for: Field Emission Properties of Cu-Filled Vertically Aligned Carbon Nanotubes Grown Directly on Thin Cu Foils
Source: Nanomaterials (Basel). 2024 Jun 6;14(11):988. doi: 10.3390/nano14110988 (PMC11174008; doi:10.3390/nano14110988)
Supplement: Supplementary file 1 [file nanomaterials-14-00988-s001.zip › nanomaterials-3030763-supplementary.pdf]

Supplementary Materials:

Field Emission Properties of Cu-Filled VACNTs Grown Directly on Thin Cu-Foils

Chinaza E. Nwanno [1], Arun Thapa [1], John Watt [2], Daniel Simkins Bendayan [1] and Wenzhi Li [1,*]

[1] Department of Physics, Florida International University, Miami, FL 33199, USA

[2] Center for Integrated Nanotechnologies, Los Alamos National Laboratory, Los Alamos, NM 87545, USA

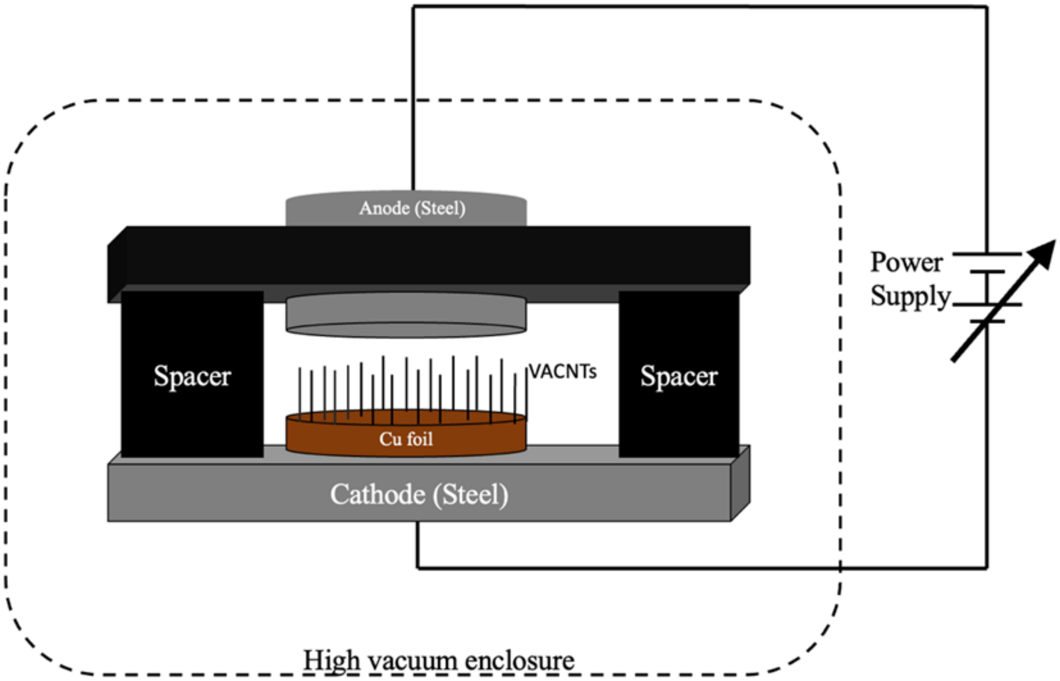

Figure S1. Schematic illustration of the setup for field emission measurements.
